# Beyond-Rule-of-Five Compounds Are Not Different: In Vitro–In Vivo Extrapolation of Female CD-1 Mouse Clearance Based on Merck Healthcare KGaA Compound Set

**DOI:** 10.3390/ph18040568

**Published:** 2025-04-14

**Authors:** Christine K. Maurer, Zhizhou Fang, Heide M. Duevel, Stephanie Harlfinger, Carl Petersson

**Affiliations:** NCE DMPK, Merck Healthcare KGaA, Frankfurter Straße 250, 64293 Darmstadt, Germany

**Keywords:** in vitro–in vivo extrapolation, metabolic clearance, microsomes, hepatocytes, fraction unbound in the incubation, PROTACs©, beyond-rule-of-five, ADME, DMPK

## Abstract

**Background:** Extrapolation of intrinsic clearance from in vitro systems such as liver microsomes or hepatocytes is an established approach to predict clearance in preclinical species and in humans. A common discussion in the literature is whether the predictive accuracy of such extrapolations is influenced by the chemotype and whether these methods are also applicable to compounds studied in early drug discovery programs. Compounds in such programs are frequently lipophilic and show low solubility and low free fraction in plasma, which may pose challenges to the extrapolation of clearance different from those of the final clinical candidates. A similar discussion has been raised about compounds residing beyond the traditional small-molecule property space, such as PROTACs© and other molecules incompatible with Lipinski’s rule-of-five. **Methods:** To further enlighten the field on these matters, we present a study comparing the predictive accuracy between mouse hepatocytes and microsomes for a set of molecules (N = 211) from the Merck Healthcare drug discovery pipeline. This set was dominated by compounds belonging to class 2 and 4 of the extended clearance classification systems (ECCS). It contained a similar proportion of molecules compliant with the Lipinski rule-of-five (N = 127) and molecules lacking such compliance (N = 84). **Results:** This study showed no or little differences in predictive accuracy nor bias between the two groups, with an average fold error close to 1, an absolute average fold error of just over 2, and around 50% being within 2-fold and >90% being within 5-fold of the predicted unbound clearance in both in vitro systems. Furthermore, no significant differences in accuracy were observed for compounds with an extremely low free fraction (down to 0.05%) in plasma. **Conclusions:** The accuracy of in vitro–in vivo extrapolation of female CD-1 mouse clearance was not affected by the physicochemical properties.

## 1. Introduction

The prediction of pharmacokinetics in humans and preclinical species is an important task in drug discovery. Hepatic metabolic clearance is a central parameter in these predictions, as clearance is a fundamental property that directly influences the dose, half-life and dosing regimen [1,2,3]. Extrapolation of hepatic metabolic clearance from in vitro systems such as liver microsomes and hepatocytes has been used for more than 20 years in drug discovery and development [4]. Several studies suggest that further modifications, empirical or mechanistic, may be required to obtain good extrapolation accuracy [5,6,7,8,9,10]. Most of these reports studied the extrapolation of human clearance and the studied molecules were clinically used molecules with proven hepatic metabolic elimination. The studied molecules were typically rule-of-five (Ro5)-compliant, i.e., they had less than two violations of the rules introduced by Lipinski [11]. Furthermore, solubility and free fraction in in vitro incubations were high for the vast majority of the studied molecules. Molecules investigated in drug discovery during the optimization of novel drug candidates and/or pharmacological tool compounds frequently deviate from these standards, as they tend to be more lipophilic, thereby limiting their solubility and frequently resulting in a low free fraction in plasma (f_u,p_) as well as in in vitro incubations. Furthermore, the rising popularity of molecules with novel mechanisms of action, such as bifunctional protein degraders (PROTACs©), has made compounds violating more than one of Lipinski’s Ro5 (bRo5) more common within the industry. Such compounds frequently have low solubility and a very low unbound fraction in plasma as well as in in vitro incubations [12,13,14,15]. The elimination pathways of this compound class have not been studied in great depth in the literature, although biliary elimination for individual compounds has been reported as well as the absence of any transporter-mediated disposition [13,15].

This study utilizes a diverse set of molecules representative of the chemistry in drug discovery programs run in our organization during the last decade. It contains a considerable portion of bRo5 compounds, many featuring high lipophilicity, low solubility and a low unbound fraction. Here, we are following up on our initial study on a smaller set of PROTACs© [12]. Therefore, the expanded compound set was suitable for assessing the impact of that physicochemical space and associated properties on the accuracy and bias of mouse clearance prediction extrapolated from two in vitro systems: microsomes and hepatocytes.

## 2. Results

### 2.1. Data Set

**Figure 1:**
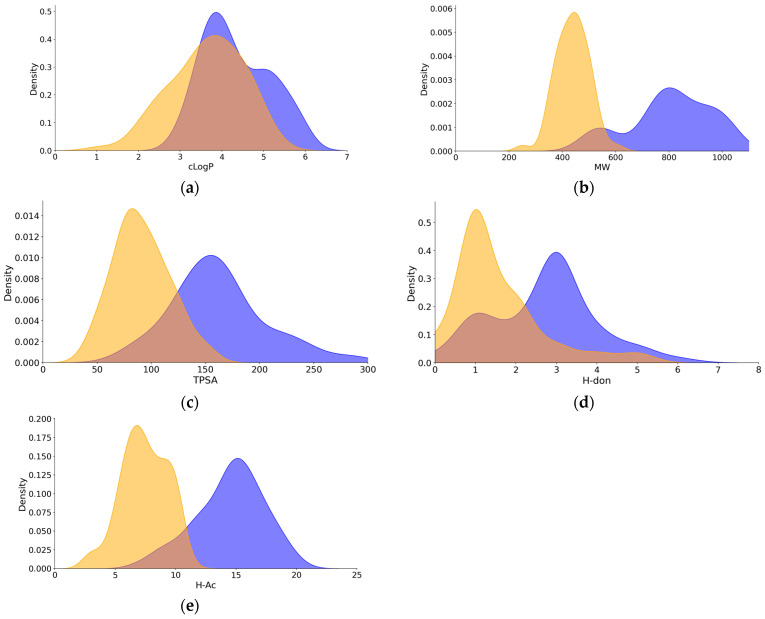
Density plots for molecular descriptors of the compound set used in this study. (**a**) Calculated logP: clogP; (**b**) molecular weight: MW; (**c**) total polar surface area: TPSA; (**d**) number of H-bond donors: HDon; (**e**) number of H-bond acceptors: HAc. Yellow color denotes the Ro5-compliant compounds, and blue color denotes the beyond-Ro5 compounds.

The evaluation set (N = 211) consisted of Ro5 (N = 127) and bRo5 (N = 84) compounds. Neutrals constituted around 70% of the compounds. The remaining ~30% were mainly bases, while other ion classes (acids, zwitterions) contributed <2%. The bRo5 group had a higher proportion of basic compounds (about 45%) compared to the Ro5-compliant compounds (about 17%). Density plots based on physicochemical properties indicated differences between the Ro5 and bRo5 compound sets in terms of molecular weight, total polar surface area, and number of H-bond donors and acceptors. The difference was less pronounced for logP (Figure 1).

### 2.2. Binding to In Vitro Systems

**Figure 2:**
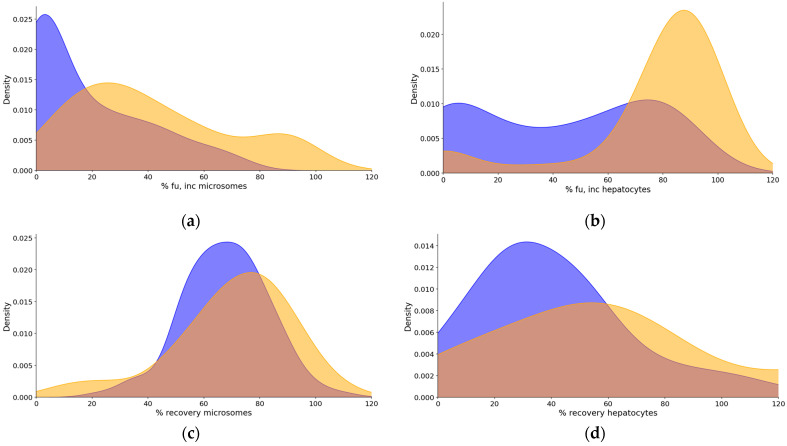
Density plots for unbound fraction (f_u_) and recovery in microsomes and hepatocytes. (**a**) % unbound fraction in microsomes: % fu, inc, microsomes; (**b**) % unbound fraction in hepatocytes: % fu, inc, hepatocytes; (**c**) % recovery in microsomes; (**d**) % recovery in hepatocytes. Yellow color denotes the Ro5-compliant compounds, and blue color denotes the beyond-Ro5 compounds.

The binding to microsomes was higher compared to hepatocytes in all compound classes (Table 1). In general, the Ro5 compounds showed a high free fraction in hepatocytes, while the bRo5 set exhibited a wider distribution, with many compounds with a low free fraction. The distribution of values was wider in microsomes for the Ro5 set, and the mean value was higher compared to the bRo5 compounds (Table 1, Figure 2). All of the above-discussed trends were more pronounced for basic compounds compared to the rest of the set. The recovery was generally high in microsomes, although a trend of lower recovery was observed for the bRo5 compounds. The recovery was frequently low in hepatocytes for the bRo5 compounds, while the Ro5 compounds showed a broad distribution of values.

The Kilford equation [16] failed to estimate a high binding of compounds and resulted in a higher free fraction in both in vitro systems compared to the measured values. The differences were low for the Ro5-compliant compounds in hepatocytes, which, in general, had a measured high free fraction (Table 1 and Table S1).

### 2.3. Extrapolation of Mouse Clearance from In Vitro Systems

#### 2.3.1. Regression Correction

**Figure 3:**
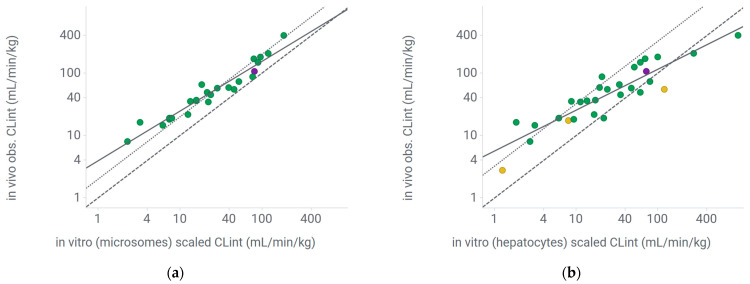
IVIVE of intrinsic clearance in mice using well-curated data set. (**a**) Based on microsomes; (**b**) based on hepatocytes. Obs.: observed, CL_int_; total intrinsic clearance; green dots: neutrals, violet dots: bases, yellow dots: acids, solid line: regression line, dashed line: line of unity, dotted line: regression line reported by Tess et al. [8].

A well-curated set of compounds (N = 29) expected to be eliminated by hepatic metabolism was selected according to the following criteria: kinetic solubility >10 µM, passive Caco-2 permeability >5 (×10^−6^ cm/s), unbound fraction (f_u_) recovery >50% in microsomes and hepatocytes, f_u_ in mouse plasma >0.5% and absence of known biliary or renal elimination. Compounds with phase II metabolism were discarded in the microsome set (N = 25). In vitro–in vivo extrapolation (IVIVE) of intrinsic clearance (CL_int_) using the well-stirred model and experimental unbound fraction data in plasma (f_u,p_)/in the in vitro systems (f_u,mic_, f_u,hep_) indicated a systematic error (Figure 3). To correct for this, the following regression equations were derived according to the approach suggested by Sternbeck et al. [6] for microsomes and hepatocytes, respectively (Figure 3): log (in vivo CL_int_) = 0.8 ×log (scaled in vivo CL_int_) + 0.6 and log (in vivo CL_int_) = 0.6 × log (scaled in vivo CL_int_) + 0.8, with CL_int_ expressed in mL/min/kg. The details of the properties of the set are available in Table S2.

#### 2.3.2. Extrapolation of Mouse Clearance from In Vitro Systems Using Experimental Unbound Fraction in the Incubation

**Figure 4:**
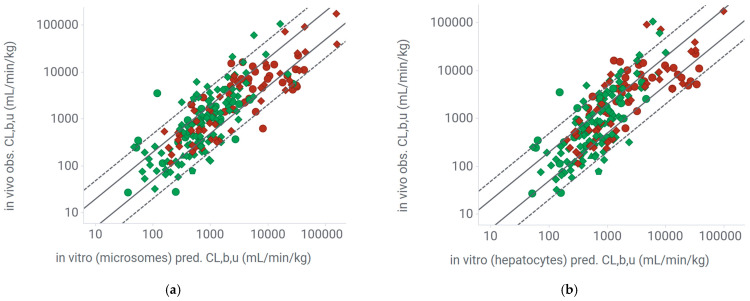
IVIVE of unbound clearance in mice for rule-of-5 (Ro5) and beyond-Ro5 (bRo5) compounds. (**a**) From microsomes; (**b**) from hepatocytes. Pred.: predicted, obs.: observed, CL_b,u_; unbound blood clearance; green dots: Ro5 compounds, red dots; bRo5 compounds; solid line: 2-fold, dashed line: 5-fold; triangles: acids, dots: bases; diamonds: neutrals, pentagons: zwitterions.

**Figure 5:**
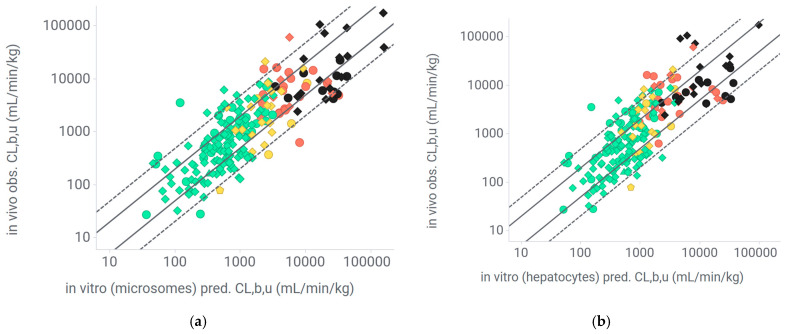
IVIVE of unbound clearance in mice for compounds with different fractions unbound in plasma. (**a**) From microsomes; (**b**) from hepatocytes. Pred.: predicted, obs.: observed, CL_b,u_; unbound blood clearance; black dots: f_u,p_ ≤ 0.2%, red dots: f_u,p_ 0.2–0.5%, yellow dots: f_u,p_ 0.5–1%, green dots: f_u,p_ > 1%; solid line: 2-fold, dashed line: 5-fold; triangles: acids, dots: bases; diamonds: neutrals, pentagons: zwitterions.

The extrapolation based on microsome data showed a low bias with an average fold error (AFE) of 1, an average absolute fold error (AAFE) of 2.2, and 53% of the compounds within 2-fold and 90% within 5-fold (Table 2, Figure 4). Similar accuracy between the Ro5 and bRo5 data sets was observed overall, with an AAFE just over 2 and around 50% of the compounds being within 2-fold and around 90% within 5-fold. Extrapolation based on hepatocytes showed a similar accuracy and no obvious bias. In this system, the subgroup of basic compounds was slightly underestimated with an AFE of 1.5, which resulted in an increased AAFE of 2.5. Overall, the performance was similar for hepatocytes and microsomes. The prediction accuracy was the same for the Ro5 and bRo5 compounds in both in vitro systems. Carefully analyzing the impact of the predictive accuracy by f_u,p_ revealed no influence in any of the in vitro systems (Figure 5 and Figure S1). A similar lack of correlation was observed for in vitro CL_int_ (Figure S1), kinetic solubility and recovery in the unbound fraction assays (Figure S2), logD and logP (Figure S3), efflux and permeability (Figure S4), H-bond donor count, and existence of a VHL-binding domain in the molecular structure (Figure S5).

A total of 15 compounds, 7% of the data set, had an observed total estimated blood clearance above the liver blood flow, i.e., 102 mL/min/kg. These compounds were underestimated by all methods as a consequence of the well-stirred model, which predicts hepatic metabolic clearance. Still, this fraction of the compounds was kept in the data set to widen the understanding of the overall accuracy. This subfraction constituted the majority of the most severely underestimated compounds, with most of them being underpredicted by more than 5-fold (Figure S6).

#### 2.3.3. Extrapolation of Mouse Clearance from In Vitro Systems Using Predicted Unbound Fraction in the Incubation

The corresponding analysis of the prediction accuracy based on the unbound fraction in the incubations calculated via the Kilford equation revealed substantial underestimations for all the examined groups (Table 3, Figure S7). This pattern was most pronounced for lipophilic compounds with high binding (bRo5 compounds) in microsomes, especially bases. These compounds showed the lowest free fractions and the largest difference between the measured and predicted values (Table 2 and Table 3). Hepatocytes showed a lower bias overall. Neutral compounds, which generally had high free fractions in hepatocytes, showed a modest underestimation (AFE 1.3) and an accuracy similar to estimates based on the measured free fraction values.

## 3. Discussion

The data set used in this study was dominated by neutral and basic compounds which was reflected in the reference set. The reference set was curated to ensure high quality extrapolation combined with a high probability of hepatic metabolism as the elimination major pathway. IVIVE of this set, in microsomes as well as hepatocytes, showed a systematic underestimation, similar to findings in other species in the literature [6,8] (Figure 3). The regression used in this study to predict the clearance of the remaining compounds showed similar slopes in microsomes and hepatocytes of 0.8 and 0.6 and intercepts of around 0.6 and 0.8, respectively. Tess et al. studied the extrapolation of clearance in male mice from in vitro systems on a group (N = 45) of neutral and basic compounds using experimentally determined free fractions in the incubation. The identified scaling factors were similar to those in this study, providing further confidence in their robustness (Figure 3, [8]).

The Ro5-compliant subset was dominated by neutrals and bases with moderate-to-high lipophilicity, which showed low unbound fractions in plasma, making renal elimination unlikely as a major elimination pathway (Figure 1) [17,18]. This also applied to the subset of bRo5 compounds, thus rendering these compounds to extended clearance classification system (ECCS) class 2 or 4, suggesting hepatic metabolism as the major clearance pathway [18]. Direct biliary elimination for PROTACs© and other bRo5 compounds has been observed in individual cases in rodents and has been discussed as a plausible elimination pathway [15].

The basis for the classification of the ECCS system is human data, and the underlying data had a modest representation of bRo5 compounds, which may limit the applicability for such compounds. Still, the absence of bias (underestimation) reported in this study suggests that metabolism was also the prevalent clearance-rate-determining step for the bRo5 subset. The same observation has been reported by Pike et al. for PROTACs©, which are generally bRo5 compounds [13]. An important limitation in the present study was the low frequency of zwitterionic and acidic compounds, i.e., members of ECCS class 1 and 3; these compound classes are generally more prone to transporter-mediated elimination, especially at a higher molecular weight [19]. Further, compounds apparently prone to metabolic pathways absent in microsomes, such as conjugation reactions, were actively removed from the compound set in an effort to focus on metabolic pathways represented in both microsomes and hepatocytes to allow for comparison between the in vitro systems. This process reduced the total number of compounds by approximately 10%, indicating a low prevalence in the studied chemical space.

A larger data set containing bRo5 compounds discussing differences on the basis of Ro5 compliance was reported by Manevski [20]. This study was based on over 2000 compounds and an extrapolation of clearance from hepatocytes. Different from this study, the predictive performance was specifically inferior for bRo5 compounds. This difference may stem from the composition of the sets, as this study was limited to ECCS classes 2 and 4. Manevski et al. also observed differences for ECCS class 2 and 4, which may be a result of the examined extrapolation approaches. The authors compensated for the lack of experimental data on the free fraction in in vitro incubations either by assuming a similar binding to fetal calf serum and mouse plasma or by assessing incubation binding by the equation suggested by Austin alone or in combination with the plasma protein binding to fetal calf serum [20]. These approximations may explain the observed underestimation of clearance for the bRo5 compounds, different to compounds with a lower molecular weight, as bRo5 compounds tend to show a low free fraction in incubations. Another difference between the studies was that Manevski et al., unlike this study, did not harmonize the strain nor the sex between the in vivo and in vitro studies. This may have impacted the overall accuracy, as differences in the expression and activity of drug-metabolizing enzymes exist between mouse strains as well as between sexes, but it is less likely to have introduced bias associated with certain physicochemical properties [21].

A total of 7% (N = 15) of the compounds in this study had an estimated blood clearance over the liver blood flow. It may appear scientifically sound and tempting to remove these compounds from the data set, as the well-stirred model used for the extrapolation from the in vitro systems is limited by the liver blood flow [4]. The estimation of clearance for these compounds was associated with an increased uncertainty due to the low area under the curve (AUC) used in their estimation. Further, the blood clearance was estimated using a generic blood-to-plasma ratio based on the ion class. There is also some variability in the reported liver blood flow in mice [13,22,23]. Hence, this compound subset is not necessarily associated with extrahepatic clearance mechanisms, even if these compounds were among the most underestimated (Figure S6). The fact that the prediction error was normally distributed for both in vitro systems even contradicts additional clearance mechanisms for this group.

Issues related to the accurate determination of extensive binding to plasma proteins as well as in incubations of bRo5 molecules have been discussed as a major challenge in the literature [15]. However, this study indicated that the accurate experimental determination of the unbound fraction in plasma and incubations was achieved, as the prediction accuracy was high, and errors were reasonably normally distributed over four orders of magnitude of unbound clearance. Our work also indicated that the industry standard method of equilibrium dialysis had a value in the quantification of very low free fractions in plasma (0.05–0.3%), as these very tightly bound compounds showed a similar prediction accuracy to compounds with higher free fractions (Figure 5). The apparent correlation between the free fraction and unbound clearance observed was most likely not of a causal nature. The major determinant of unbound clearance is lipophilicity [24]. Lipophilicity is, however, highly correlated with the free fraction in plasma, causing the illusion that a low free fraction causes a high unbound clearance.

Basic compounds showed a slight bias, with AFE 1.5., when the extrapolation was carried out based on hepatocytes. The bias was observed regardless of Ro5 compliance. We were unable to link this to any distinct property or ADME endpoint. It is plausible that a different regression factor was required, as the reference set used was dominated by neutral compounds, but the fact that no bias was observed for microsomes made it difficult to rationalize further.

Stronger binding to microsomes compared to hepatocytes was observed in all the compound groups. This observation can primarily be attributed to the higher phospholipid levels in the microsomal incubation at the protein/cell concentrations employed in the experiments (see Section 4). The recovery was substantially lower in hepatocytes, especially for the bRo5 set, which we attributed to adsorption onto device surfaces and/or solubility limitations, whereas in microsomes, the phospholipid interactions were strong enough to prevent the loss of compounds due to unspecific binding to plastic. The fact that the predictive accuracy of the clearance extrapolation was similar regardless of the recovery suggests that the system reached equilibrium with regards to unspecific binding.

The Kilford equation underestimated binding for strongly bound compounds in both systems (Table 1). This is in line with the original publication suggesting an applicability domain below logD 2.5 [16] and with our recent findings based on the IVIVE analysis of a small set of PROTACs© [12]. This pattern of underestimation for lipophilic highly bound compounds is also in agreement with a cross-company study by Winiwarter et al. [25]. The limited accuracy of the Kilford equation resulted in limited prediction accuracy and strong underestimation for extrapolation using microsomes. The differences compared to the measured values were less pronounced in hepatocytes, especially for the Ro5 set, due to the inherently low binding measured for this subset.

In this study, we demonstrated comparable accuracy for the extrapolation of mouse clearance from hepatocytes as well as microsomes irrespective of the Ro5 classification of the compounds. This was under the assumption that the blood-to-plasma ratio (R_b_) was 1 for the majority of the compounds in the set. The obtained accuracy may indeed reflect the fact that most of the compounds had a ratio close to this number; however, an experimental assessment would eliminate this assumption and may further increase the accuracy. Further, a similar accuracy was obtained for compounds with a low free fraction in plasma, down to 0.05%, to compounds with a high free fraction, which adds confidence for standard methods in quantifying low free fractions.

The predicted endpoint, unbound clearance, is proportional to unbound exposure, which, in turn, is proportional to the pharmacologically relevant exposure in the absence of active transport [26]. Given the relatively small compound test set used in the present study, we would welcome the utilization of similar methods for larger data sets and other preclinical species to provide further confidence in our results. Similar results from additional analyses would further strengthen the value of in vitro systems, thereby reducing the need for animal experiments, and they would provide a means of increasing the speed of optimization efforts for bRo5 compounds like PROTACs©.

## 4. Materials and Methods

The materials used in this paper and the method descriptions for the CaCo-2 and kinetic solubility assays are presented in the Supporting Information.

### 4.1. Data Set

The data set used in this study consisted of compounds for which in-house female CD-1 mouse IV PK data were available. Compounds with a free fraction below the limit of quantification in mouse plasma were removed (N = 10). Compounds with a 2-fold or higher predicted clearance from hepatocytes compared to microsomes were removed to ensure comparability between the two systems (N = 25). Further confidence in the comparability between the systems was obtained from the correlation of the predicted intrinsic clearance (Figure S8). The pre-treatment resulted in a data set of 211 molecules. This group was divided according to the rule-of-5 (Ro5) leading to two subsets consisting of 127 Ro5-compliant molecules and 84 compounds that violated more than 1 of the bespoke criteria [11].

### 4.2. Determination of CL_int_ in Mouse Microsomes

Microsomal stability was assessed on a liquid handling platform (Tecan Freedom EVO-2 200 MCA384, Männedorf, Switzerland) using pooled female CD-1 mouse microsomes. Compounds (1 µM final) were pre-incubated with microsomes (final 0.5 mg/mL) for 5 min at 37 °C in phosphate buffer (50 mM, pH 7.4), which was followed by the start of the incubation by the addition of cofactor NADPH (final 1.5 mM). The final dimethyl sulfoxide (DMSO) concentration did not exceed 0.2% (*v*/*v*). Incubations were carried out in singlicate. Aliquots were taken at time points of 0, 5, 10, 20, and 30 min. Additionally, two control samples (0 and 30 min) lacking the addition of NADPH were included in order to flag compounds with non-microsomal metabolism. After incubation, the samples were quenched by adding sample aliquots from four different compound incubations to a two-fold volume of acetonitrile (ACN) for bioanalytical cocktailing designed to avoid analytical interference. After centrifugation, the supernatants were analyzed by a standard reversed-phase liquid chromatography assay coupled to tandem mass spectrometry (UHPLC-MS/MS) (for details, see the Supplementary Materials).

The data were subjected to post-acquisition analysis by the Analyst software (version 1.7.2), and the percentage of compound remaining (defined as the ratio of the peak area at a given time point and that at the 0 min time point) was plotted against the incubation time. The first-order elimination rate constant (k_el_) was derived by non-linear regression using Biorails version 5.10.22/Morphit version 8.0 (The Edge Software Consultancy, Guildford, UK), and CL_int_ values (expressed in µL/min/mg) were obtained by normalizing k_el_ with the microsome concentration.

### 4.3. Determination of CL_int_ in Mouse Hepatocytes

Hepatocyte stability was assessed, as described previously [12], on a liquid handling platform (Hamilton Microlab Vantage, Bonaduz, Switzerland) using cryopreserved pooled female CD-1 mouse hepatocytes in suspension. Viability was determined in a Neubauer chamber by trypan blue staining and was always above 70%. Compounds (1 µM final) were incubated with hepatocytes at a cell density of 0.2 × 106 cells/mL in Krebs–Henseleit buffer (pH 7.4). The final DMSO concentration did not exceed 1% (*v*/*v*). Incubations were carried out in duplicate at 37 °C under an atmosphere of 5% CO_2_ and 95% humidity. Aliquots were taken at time points of 0, 10, 20, 40, 60, and 90 min and quenched with a two-fold volume of an ACN solution containing 1.5 µM of an internal standard. The supernatants were further diluted to a ratio of 1:3 (*v*/*v*) with supernatants from other compound incubations for bioanalytical cocktailing designed to avoid analytical interference. The samples were analyzed by UHPLC-MS/MS (for details, see the Supplementary Materials).

For quantitation with the Analyst software (version 1.7.2), MS peak area ratios of the compounds and an internal standard were calculated and transformed to nanomolar concentrations determined based on a four-point calibration comprising 1.2% to 150% of the incubation concentration. Calibration standards were prepared using heat-inactivated (60 °C for at least 15 min) hepatocytes as a biomatrix and processed in duplicate, as described for the test samples.

For data analysis, the concentration data were plotted against the incubation time, the first-order k_el_ was determined by non-linear regression using Biorails version 5.10.22/Morphit version 8.0 (The Edge Software Consultancy, Guildford, UK), and CL_int_ values (expressed in µL/min/10^6^ cells) were obtained by normalizing k_el_ with the hepatocyte concentration.

### 4.4. Prediction of f_u,mic_ and f_u,hep_ by the Kilford Equations

The free fractions in the microsome and the hepatocyte incubations were predicted using equations 4 and 5 from Kilford et al. [16], respectively. The microsomal protein concentration, P, was 0.5, and the VR was 0.001 (given a cell density of 0.2 × 10^6^ cells/mL). The calculated logP (clogP) value was used for the basic ion class, the clogD_7.4_ for the acidic and neutral ion classes. The clogP and clogD_7.4_ values were calculated using GALAS (Global Adjusted Locally According to Similarity) method within Percepta (ACD Labs, version 14.50, Toronto, ON, Canada) retrained with inhouse experimental logD_7.4_ and pK_a_ data.

### 4.5. Determination of Unbound Fraction in Plasma (f_u,p_), in the Microsome Incubation (f_u,mic_), and in the Hepatocyte Incubation (f_u,hep_)

The free fraction in mouse plasma, in the microsome incubation, and in the hepatocyte incubation was determined using rapid equilibrium dialysis (RED) in Teflon-coated 96-well RED devices. Test compounds were added as DMSO stocks to the donor matrix at 0.5 µM (final DMSO concentration of 0.5%) and dialyzed against phosphate buffer (70 mM, pH 7.4) in the receiver compartment for 4 h at 37 °C.

The donor matrices differed for the various unbound fractions. For f_u,p_, serum was used as a surrogate, as the in-house data showed that the f_u_ values did not differ between the serum and plasma. Mouse serum was dialyzed overnight to exchange the carbonate with a phosphate buffer and was then used as a donor matrix in the experiment (50% serum in 70 mM phosphate buffer, pH 7.4). For f_u,mic_, human liver microsomes were used as a surrogate for mouse microsomes [27] as a donor matrix in the experiment (0.8 mg protein/mL in 70 mM phosphate buffer, pH 7.4). For f_u,hep_, mouse hepatocytes were inactivated by incubating overnight at room temperature and then used as a donor matrix in the experiment (0.2 million cells/mL in 70 mM phosphate buffer, pH 7.4).

Samples were taken from both compartments after 4 h of incubation and analyzed via UHPLC-MS/MS. The unbound fraction, fu, was calculated as follows:

$$f_{u}=\frac{c_{receiver}}{c_{donor}}$$

The f_u_ values were interconverted from one matrix concentration to another using the following formula:

$$f_{u2}=\frac{f_{u1}}{\frac{M2}{M1}-f_{u1}\cdot \left(\frac{M2}{M1}-1\right)}$$

where f_u2_ at matrix concentration M2 can be calculated from the f_u1_ value at matrix concentration M1.

The recovery was calculated to check the mass balance after the experiment by adding the total compound levels in both the donor and receiver compartments.

### 4.6. Pharmacokinetic Studies in Mice

Pharmacokinetic studies were carried out after approval by the internal animal welfare board and in compliance with the local and international animal welfare regulations similarly to a method described previously [12]. Generally, three or twelve CD-1 mice per study were dosed intravenously (0.2–1 mg/kg) using solution vehicles, predominantly 40% PEG 200 in 1% DMSO. For the majority of the studies, blood samples were taken retro-orbitally under inhalation of anesthesia (0.1, 0.5, 1, 2, 4, 6, and 24 h). For the remaining studies, blood sampling was performed both retro-orbitally (0.1, 0.5, 1, 2, and 24 h) and from the saphenous vein (4 and 6 h) (N = 51), from the dorsal metatarsal vein (N = 34), or submandibularly (N = 12). Full details regarding the sampling methods for each compound are given in Tables S1 and S2. Plasma was obtained by centrifugation (10,000× *g*; 4 °C; 5 min) and stored at −20 °C until UHPLC-MS/MS analysis. By the addition of an ACN solution containing internal standard, the proteins were precipitated and dissevered by centrifugation. The concentration of the respective molecules studied was determined by a standard UHPLC-MS/MS method (generic method similar to the method described for the determination of CL_int_ using experimentally determined compound MS tuning parameters). Plasma concentrations were determined for each time point sampled. Clearance was estimated from the AUC by non-compartmental analysis, which was performed using WinNonlin^®^ (Princeton, NJ, USA). The geometric mean was used when more than one clearance value from different studies was available.

### 4.7. IVIVE of Mouse CL_int_ from In Vitro Systems

For regression correction, a set of compounds (N = 29) expected to be eliminated by hepatic metabolism and with properties making high-quality extrapolation likely was defined by the following criteria: kinetic solubility >10 µM, passive Caco-2 permeability >5 (×10^−6^ cm/s), f_u_ recovery >50% in microsomes and hepatocytes, f_u_ in mouse plasma >0.5%, and an absence of known biliary or renal elimination. Compounds with phase II metabolism were removed from the microsome set (N = 25). IVIVE using the well-stirred model and experimental fraction unbound data in plasma/in the in vitro systems, R_b_ assumed to be 1 for bases/neutrals/zwitterions and 0.55 for acids indicated a systematic error, which was corrected by the model suggested by Sohlenius-Sternbeck et al. [6]. The following regression equations were derived for microsomes and hepatocytes, respectively: log predicted CL_int_ = 0.8 * log scaled CL_int_ + 0.6 and log predicted CL_int_ = 0.6 * log scaled CL_int_ + 0.8, with CL_int_ expressed in mL/min/kg. Full details of the properties of the set are given in Table S2.

According to Sohlenius-Sternbeck et al. [6], the following equations were used for IVIVE:

$$scaled\;CLint,b=in\;vitro\;CLint*\frac{microsomes/hepatocytes}{gram\;of\;liver}*\frac{liver\;weight}{body\;weight}*\frac{fu,p}{fu,inc*Rb}\;$$

The physiological scaling factors, taken from Simcyp^TM^ version 13/1 (Certara), were 48 mg of microsomal protein per gram of liver (MPPGL), 101.7 × 10^6^ hepatocytes per gram of liver (HPGL), a liver weight of 1.46 g, and a body weight of 0.025 kg. For microsomes and hepatocytes, f_u,mic_ and f_u,hep_, were used as the unbound fraction in the incubation (f_u,inc_), respectively.

predicted CLint,b=10^(log scaled CLint∗slope+intercept)

For microsomes, a slope of 0.8 and an intercept of 0.6 were used, and for hepatocytes, a slope of 0.6 and an intercept of 0.8, according to the regression equations derived above.

$$predicted\;CL,b=\frac{pred.\;CLint,b*QH}{pred.\;Clint,b+QH}$$

For female CD-1 mice, a liver blood flow (Q_H_) of 101.7 mL/min/kg was used (Simcyp^TM^ version 14, Certara).

$$observed\;CL,b=\frac{obs.\;CLp}{Rb}$$

$$observed\;CLint,b=\frac{QH*obs.\;CL,b}{QH-obs.\;CL,b}$$

All unbound clearance terms were calculated by dividing the respective clearance by f_u,p_.

$$predicted/observed\;CL,b,u=\frac{pred./obs.\;CL,b}{fu,p}$$

The above-described approach was applied for the IVIVE of clearance of the data set analyzed in this study. Additionally, in order to evaluate the impact of the Kilford calculations as a surrogate for the experimentally determined free fraction in the in vitro systems, clearance was extrapolated using the Kilford equations [16] using the same regression coefficients.

### 4.8. Statistics

For performance evaluation of IVIVE, the following statistical analyses were carried out.

From the IVIVE plots, a measure of the bias, expressed as the AFE, that is, the geometric mean fold error, was calculated as follows [28]:

$$AFE=10^{\frac{1}{N}\times \sum^{LogY_{Pred}/Y}}$$

The AAFE, a measure of the precision, was calculated using the following equation [28]:

$$AAFE=10^{\frac{1}{N}\times \sum^{\left|LogY_{Pred}/Y\right|}}$$

## Figures and Tables

**Table 1:** Mean and standard deviation of free fraction and recovery in microsomes and hepatocytes assessed by equilibrium dialysis and the Kilford equation. B: base; N: neutral; f_u,mic/hep_: unbound fraction in microsomes/hepatocytes; Ro5: compounds with <1 violation; bRo5: compounds with >1 violation.

Ion Class ^1^/Ro5	No.	f_u,mic_	Microsomes Recovery (%)	Kilford f_u,mic_	f_u,hep_	Hepatocytes Recovery (%)	Kilford f_u,hep_
All Ro5	127	0.42 ± 0.30	74 ± 20	0.90 ± 0.03	0.85 ± 0.16	82 ± 48	0.97 ± 0.01
All bRo5	84	0.18 ± 0.21	61 ± 12	0.87 ± 0.03	0.47 ± 0.32	46 ± 30	0.96 ± 0.01
N Ro5	103	0.42 ± 0.30	74 ± 20	0.89 ± 0.02	0.86 ± 0.14	81 ± 45	0.97 ± 0.01
N bRo5	45	0.26 ± 0.23	62 ± 9	0.88 ± 0.02	0.56 ± 0.31	50 ± 27	0.97 ± 0.01
B Ro5	21	0.37 ± 0.28	68 ± 21	0.89 ± 0.03	0.79 ± 0.22	77 ± 55	0.97 ± 0.01
B bRo5	38	0.07 ± 0.10	59 ± 14	0.86 ± 0.03	0.35 ± 0.29	37 ± 25	0.96 ± 0.01

^1^ Two acids and two zwitterions included in the group “all” but not analyzed separately.

**Table 2:** Accuracy of extrapolation of mouse clearance from microsomes and hepatocytes using measured free fraction in incubations. Ro5: rule-of-5-compliant; AFE: average fold error; AAFE: absolute average fold error; bRo5: beyond-Ro5; B: base; N: neutral; Mic: microsomes; Hep: hepatocytes.

Ion Class ^1^/System/Ro5	No.	AFE	AAFE	% 2-Fold	% 5-Fold
All Mic	211	1.0	2.2	53	90
All Hep	211	1.2	2.3	46	88
All Mic Ro5	127	1.1	2.3	53	87
All Mic bRo5	84	0.9	2.2	54	94
All Hep Ro5	127	1.2	2.4	44	87
All Hep bRo5	84	1.3	2.3	50	89
N Mic Ro5	103	1.1	2.2	53	90
N Mic bRo5	45	0.8	2.1	53	100
N Hep Ro5	103	1.2	2.3	43	89
N Hep bRo5	45	1.1	2.2	58	89
B Mic Ro5	21	1.1	2.5	52	81
B Mic bRo5	38	0.9	2.3	55	87
B Hep Ro5	21	1.5	2.5	57	81
B Hep bRo5	38	1.4	2.5	39	89

^1^ Group “all” included two acids and two zwitterions that were not analyzed separately.

**Table 3:** Accuracy of extrapolation of mouse clearance from microsomes and hepatocytes using free fraction estimated by the Kilford equation. Ro5: rule-of-5-compliant; AFE: average fold error; AAFE: absolute average fold error; bRo5: beyond-Ro5; B: base; N: neutral; Mic: microsomes; Hep: hepatocytes.

Ion Class ^1^/System/Ro5	No.	AFE	AAFE	% 2-Fold	% 5-Fold
All Mic	211	3.4	4.3	35	61
All Hep	211	1.8	2.8	38	80
All Mic Ro5	127	2.3	3.3	40	69
All Mic bRo5	84	6.0	6.5	26	50
All Hep Ro5	127	1.3	2.5	43	86
All Hep bRo5	84	2.7	3.5	32	71
N Mic Ro5	103	2.3	3.2	42	72
N Mic bRo5	45	3.5	4.1	40	67
N Hep Ro5	103	1.3	2.4	43	88
N Hep bRo5	45	2.1	3.4	47	73
B Mic Ro5	21	2.8	3.7	33	57
B Mic bRo5	38	11.2	11.5	11	29
B Hep Ro5	21	1.7	2.6	48	76
B Hep bRo5	38	3.9	4.2	13	68

^1^ Group “all” included two acids and two zwitterions that were not analyzed separately.

## Data Availability

All experimental and calculated data used as inputs for and resulting from the IVIVE and related analyses based on the general data set and on the regression compound set are available in the Supporting Information in Table S1 and Table S2, respectively. Structural information is included for publicly available molecules, while structural data are not disclosed for unpublished compounds proprietary to the company. Molecular weights are given and rounded to the nearest ten.

## Supplementary Materials

The following supporting information can be downloaded at: https://www.mdpi.com/article/10.3390/ph18040568/s1, Table S1: Overview data set, Table S2: Overview regression compounds. Supporting information containing the following tables and figures: Table S3: Overview of materials, Table S4: Gradient for UHPLC analysis (CL_int_), Table S5: Gradient for UHPLC analysis (kinetic solubility), Figure S1: Box plots—Impact of the fraction unbound in plasma and the in vitro intrinsic clearance on clearance prediction accuracy, Figure S2: Box plots—Impact of kinetic solubility and recovery in the fraction unbound in microsomes/hepatocytes assay on clearance prediction accuracy, Figure S3: Box plots—Impact of calculated logD at pH 7.4 (clogD 7.4) and clogP on clearance prediction accuracy, Figure S4: Box plots—Impact of CaCo-2 efflux ratio and passive permeability on clearance prediction accuracy, Figure S5: IVIVE of unbound clearance in mice highlighting compounds with number of H-bond donors > 4 and containing VHL binder, Figure S6: IVIVE of unbound clearance in mice highlighting compounds with blood clearance above the liver blood flow, Figure S7: IVIVE of unbound clearance in mice for rule-of-5 and beyond-Ro5 compounds using fraction unbound in the incubations estimated by the Kilford equation, Figure S8: Correlation of intrinsic clearances predicted from hepatocytes *vs*. microsomes, Figure S9: IVIVE of unbound clearance in mice highlighting that the different blood sampling techniques applied in in vivo PK studies have no impact on clearance extrapolation accuracy.

## Abbreviations

The following abbreviations are used in this manuscript:

AAFE	Average absolute fold error
ACN	Acetonitrile
ADME	Absorption, distribution, metabolism, excretion
AFE	Average fold error
AUC	Area under the curve
B	Base
CL_int_	Intrinsic clearance
CL_b,u_	Unbound blood clearance
clogD 7.4	Calculated logD at pH 7.4
clogP	Calculated logP
DMPK	Drug metabolism and pharmacokinetics
DMSO	Dimethyl sulfoxide
ECCS	Extended clearance classification system
f_u,hep_	Unbound fraction in the hepatocyte incubation
f_u,mic_	Unbound fraction in the microsome incubation
fu_,p_	Unbound fraction in plasma
HAc	H-bond acceptor
HDon	H-bond donor
Hep	Hepatocytes
HPGL	Hepatocytes per gram of liver
IV	Intravenous
IVIVE	In vitro–in vivo extrapolation
k_el_	Elimination rate constant
Mic	Microsomes
MPPGL	Microsomal protein per gram of liver
MW	Molecular weight
N	Neutral
NADPH	Nicotinamide adenine dinucleotide phosphate hydrogen
PROTAC©	Proteolysis-targeting chimera
PK	Pharmacokinetics
Q_H_	Hepatic blood flow
R_b_	Blood-to-plasma partition coefficient
RED	Rapid equilibrium dialysis
TPSA	Total polar surface area
UHPLC-MS/MS	Ultra-high-performance liquid chromatography–tandem mass spectrometry
VHL	Von Hippel–Lindau
