# Supplementary material for: Beyond-Rule-of-Five Compounds Are Not Different: In Vitro–In Vivo Extrapolation of Female CD-1 Mouse Clearance Based on Merck Healthcare KGaA Compound Set
Source: Pharmaceuticals (Basel). 2025 Apr 14;18(4):568. doi: 10.3390/ph18040568 (PMC12029992; doi:10.3390/ph18040568)
Supplement: Supplementary file 1 [file pharmaceuticals-18-00568-s001.zip › Supporting information_Beyond-rule-of-five compounds are not different.pdf]

pharmaceuticals

*Article*

# Beyond-Rule-of-five Compounds Are Not Different: In Vitro–In Vivo Extrapolation of Female CD-1 Mouse Clearance Based on Merck Healthcare KGaA Compound Set

**Christine K. Maurer, Zhizhou Fang, Heide M. Duevel, Stephanie Harlfinger and Carl Petersson ***

NCE DMPK, Merck Healthcare KGaA, Frankfurter Straße 250, 64293 Darmstadt, Germany; christine.maurer@merckgroup.com (C.K.M.); zhizhou.fang@merckgroup.com (Z.F.); heide.duevel@merckgroup.com (H.M.D.), stephanie.harlfinger@merckgroup.com (S.H.)

* Correspondence: carl.petersson@merckgroup.com; Tel.: +49-151-1454-3955

# Supporting information

# 1. Materials

**Table S3.** Overview of materials.

| Reagent | Provider |
|---|---|
| Pooled female CD-1 mouse microsomes, lots Corning 7055001, Corning 318003, BioIVT DVO | Corning / BioIVT |
| Cryopreserved pooled female CD-1 mouse hepatocytes in suspension, pools of 36 to 63 donors, lots TBF, QPT, TJH, RQU, DKL, UTF | BioIVT |
| Krebs-Henseleit buffer | BioIVT |
| Thawing medium for hepatocytes | BioIVT |
| DMSO | Millipore Sigma |
| Trypan blue | Millipore Sigma |
| Acetonitrile | Millipore Sigma |
| Ammonium formate | Fluka |
| Formic acid | Millipore Sigma |

# 2. Methods

*2.1. Ultra-high performance liquid chromatography coupled with tandem mass spectrometry (UHPLC-MS/MS) method used for determination of intrinsic clearance ($CL_{int}$)*

UHPLC method:

Instrument: Waters i-class UHPLC

Eluent A: Water with 0.1 % (v/v) formic acid, 10 mM ammonium formate

Eluent B: Acetonitrile

Gradient:

**Table S4.** Gradient for UHPLC analysis

| Time | Flow Rate | A [%] | B [%] | Curve |
|---|---|---|---|---|
| Initial | 0.8 | 100 | 0 | 6 |
| 0.1 | 0.8 | 100 | 0 | 6 |
| 0.8 | 0.8 | 0 | 100 | 6 |
| 1 | 0.8 | 0 | 100 | 6 |
| 1.02 | 0.8 | 100 | 0 | 6 |
| 1.4 | 0.8 | 100 | 0 | 6 |

Column: Acquity UPLC BEH C18 Column (2.1 x 50 mm)

Temperature column oven: 50 °C

Temperature sample manager: 10 °C

Temperature sample organizer: 10 °C

Injection volume: 4 μL

MS/MS method:

Instrument: Sciex API 6500, software Analyst

Ionization mode: electrospray, positive (ESI+)

Scan type: multiple reaction monitoring (MRM)

Ion source temperature: 600 °C

Mass transitions and tuning parameters were determined experimentally for each compound.

*2.2. Kinetic solubility*

The kinetic solubility (KSOL) determination is carried out in a 96-well plate filtration format where the sample (2 µL of sample stock solution 10mM DMSO) is combined to 98 µL of 20 mM buffer phosphate pH 7.4. The filtration plate is then incubated and agitated at 250 rpm for 120 minutes. After stirring, the plate is centrifuged at 2500 rpm for 3 minutes, allowing all solvent to be collected in the lower microplate. Next to avoid precipitation, 50 µL of the filtered solution is diluted with 50 µL of 2% DMSO in pH 7.4 buffer solution to yield a 100 µM sample solution ready for H(U)PLC analytics.

For the standard, each compound is prepared by combining 2 µL of the sample stock solution 10mM DMSO to 198 µL Acetonitrile/Methanol/Eluant A (1:1:1:2 v/v/v).

For the evaluation, wavelength with maximum sensitivity is to be selected. The kinetic solubility is determined according to following formulas, whereas S is the targeted value for KSOL.

$$L = \frac{area_{sample} \times concentration_{standard} \times dilution\ factor_{sample}}{area_{standard} \times dilution\ factor_{standard}}$$

$$S = \frac{L\ \times molar\ weight}{1000}$$

with: L= Concentration of solved compound in buffer [µg/ml] and S= Molar concentration of solved compound in buffer [mol/L]

Chromatographic conditions: Column Waters XBridge Column C8 3.5µm (Waters Cat No 186003053); Column temperature 37°C; Autosampler Room temperature (approx. 25°C); Injection volume 10 µL (HPLC) / 3µL (UPLC); Wavelength 220 nm, 254 nm, 290 nm. Eluant A: 1 mL Formic Acid + 999 mL Ultrapure Water; Eluant B: 1 mL Formic Acid + 999 mL Acetonitrile.

**Table S5.** Gradient for UHPLC analysis

| Time | Eluent A (%) | Eluent B (%) | Flow (ml/min) |
|---|---|---|---|
| 0.0 | 90 | 10 | 1.7 |
| 0.3 | 90 | 10 | 1.7 |
| 2.0 | 10 | 90 | 1.7 |
| 2.75 | 10 | 90 | 1.7 |
| 2.76 | 90 | 10 | 2.5 |
| 4.0 | 90 | 10 | 2.5 |

Reagents and Materials: Phosphate Buffer 20mM pH 7.4: EMS, Art No. 11601-40; DMSO Merck Art. No. 102950; Acetonitrile Merck, Art. No. 100030, Methanol Merck Art. No. 106007; Formic Acid Merck Art.No.100264; Acetonitrile Merck Art. No.10030; Filtration Plate Millipore MultiScreen HTS HV 0.45uM; 96 Well Millipore Art. No. MSHVN4510; Column Waters XBridge Column C8 3.5μm (Waters Cat. No. 186003053); HPLC vials VWR Cat. No. 5480028; Micro-Inserts VWR Cat. No. 548-0006; Screw caps VWR Cat. No. 548-0788.

### 2.3. Caco-2 assay

The Caco-2 assays were performed in a transwell assay setup using Caco-2 cells (TC7 clone). The Caco-2 cells were seeded into the apical wells (125000 cells per well) in DMEM (Dulbecco's Modified Eagle's Medium) with 20% FBS (fetal bovine serum) into Corning 24-well transwell plates and cultured for 14-21 days. Prior to the experiment, the plates were washed with HBSS (Hanks' Balanced Salt Solution). Apparent permeability $P_{app}$ was determined from apical-to-basolateral ($P_{app,AB}$) by adding 1 μL of the test compound in HBSS into the apical compartment and HBSS in the basolateral compartment, and vice versa for $P_{app,BA}$ from basolateral-to-apical. Apical volumes were 250 μL, basolateral volumes were 750 μL. DMSO content was <1 % (v/v) in all wells. Monolayer tightness was controlled using melagatran as tightness marker.

Samples were taken from both compartments at timepoint $t_0$ and after 2 hours incubation at 37 °C in 5% $CO_2$ and 100% humidity, and analyzed via UHPLC-MS/MS. The apparent permeability $P_{app}$ was calculated as follows:

$$P_{app} = \frac{\Delta c_{rec}}{\Delta t} \cdot \frac{V_{rec}}{A\ x\ c_{don,0}}$$

Where $\Delta c_{rec}/\Delta t$ is the change of concentration in the receiver compartment over the incubation time (i.e. 2 hours), $V_{rec}$ is volume of the receiver compartment, $c_{don,0}$ is the concentration in the donor compartment at time $t_0$, and A is the surface of the membrane on which the cells grew (i.e. 0.33 cm$^2$).

Mass balance was checked after the experiment by determining the recovery after the experiment:

$$Recovery = \frac{n_{don,end} + n_{don,rec}}{n_{don,0}} = \frac{c_{don,end} \cdot V_{don} + c_{rec,end} \cdot V_{rec}}{c_{don,0} \times V_{don}}$$

The efflux ratio (ER) describes affinity towards efflux transporters:

$$ER = \frac{P_{app,BA}}{P_{app,AB}}$$

The geometric mean of $P_{app,AB}$ and $P_{app,BA}$ was used as passive permeability $P_{app,pass}$.

## 3. Figures

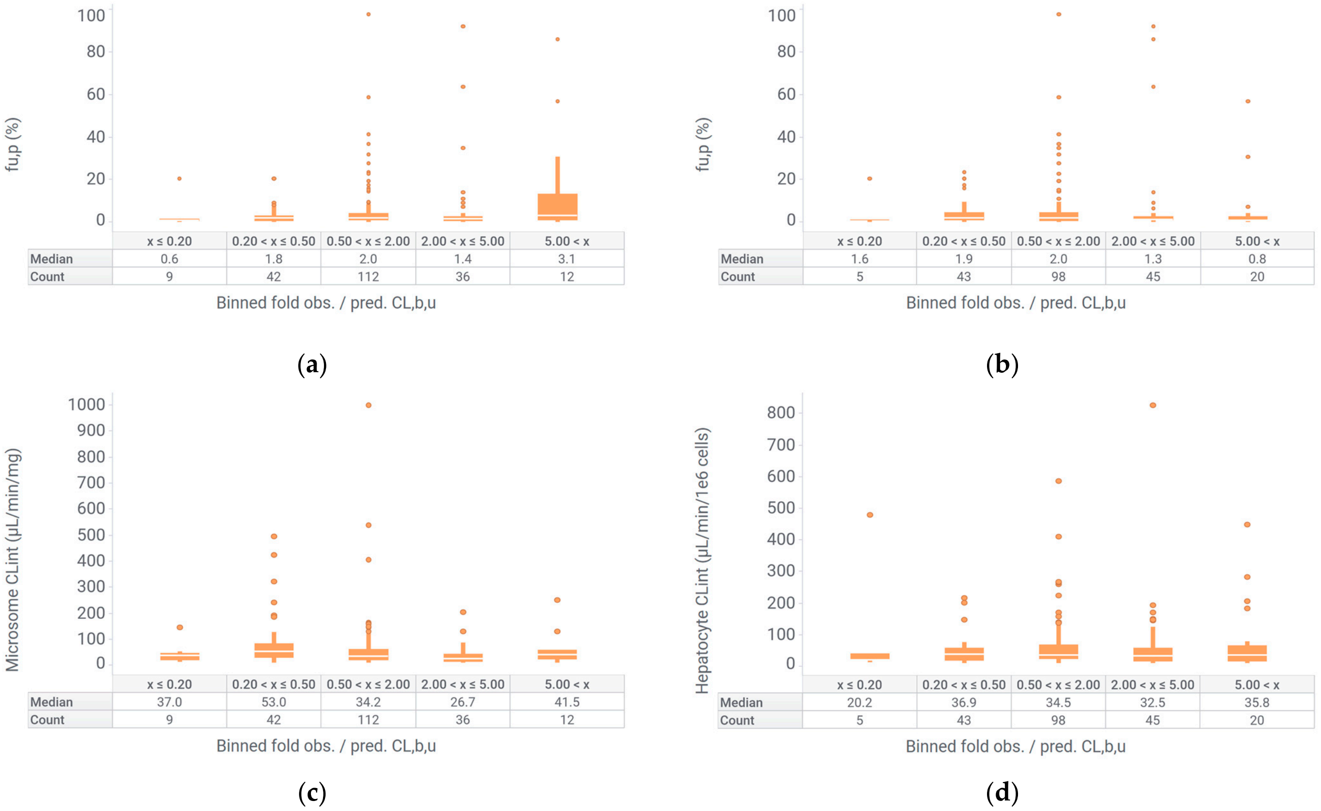


**Figure S1.** Box plots – Impact of the fraction unbound in plasma and the *in vitro* intrinsic clearance on clearance prediction accuracy. (**a**) and (**c**) Microsomes. (**b**) and (**d**) Hepatocytes. Obs.: observed, pred.: predicted, $CL_{b,u}$: unbound blood clearance, $f_{u,p}$: fraction unbound in plasma, $CL_{int}$: intrinsic clearance, white line: median.

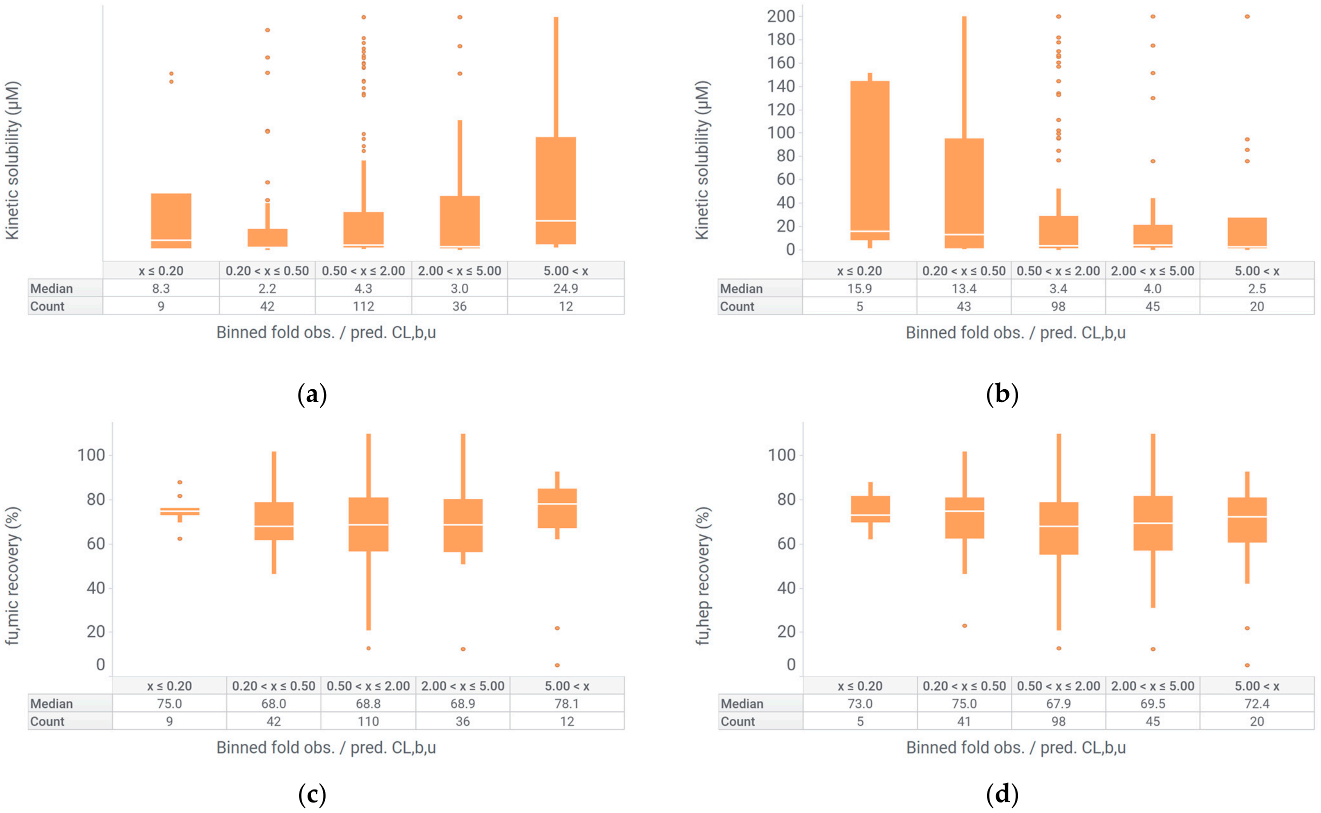


**Figure S2.** Box plots – Impact of kinetic solubility and recovery in the fraction unbound in microsomes/hepatocytes assay on clearance prediction accuracy. (**a**) and (**c**) Microsomes. (**b**) and (**d**) Hepatocytes. Obs.: observed, pred.: predicted, $CL_{b,u}$: unbound blood clearance, $f_{u,mic}$ / $f_{u,hep}$: fraction unbound in microsomes / hepatocytes, white line: median.

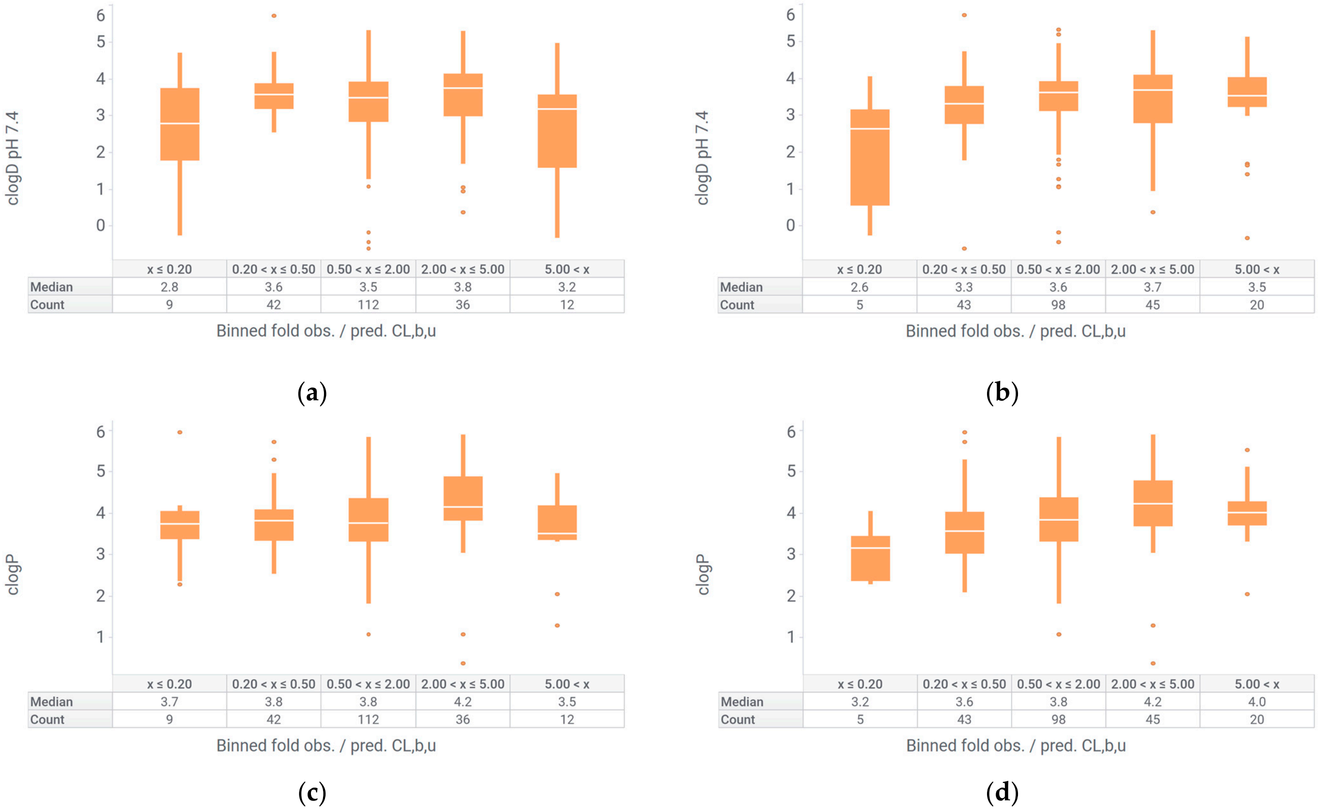


**Figure S3.** Box plots – Impact of calculated logD at pH 7.4 (clogD 7.4) and clogP on clearance prediction accuracy. (**a**) and (**c**) Microsomes. (**b**) and (**d**) Hepatocytes. Obs.: observed, pred.: predicted, $CL_{b,u}$: unbound blood clearance, white line: median.

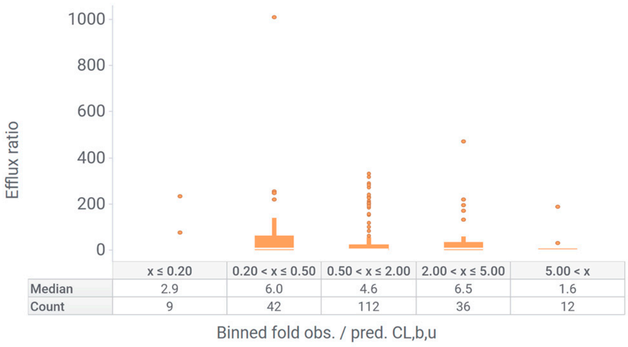


(**a**)

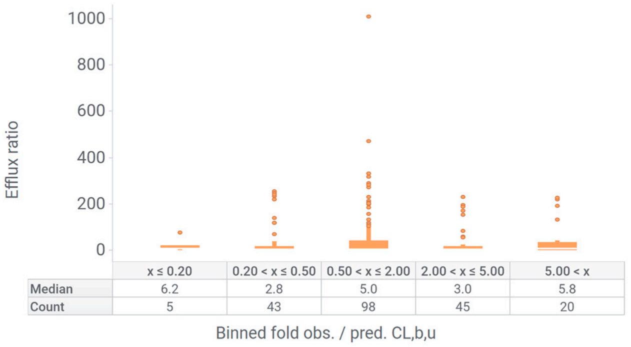


(**b**)

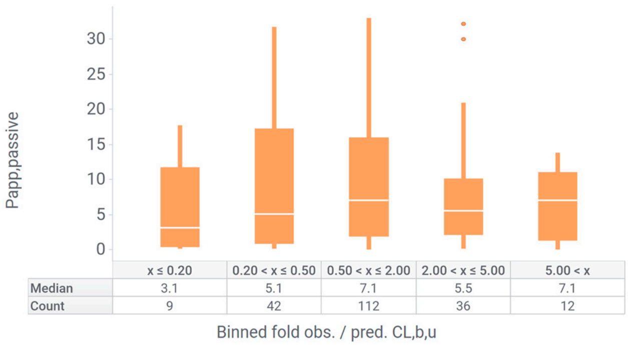


(**c**)

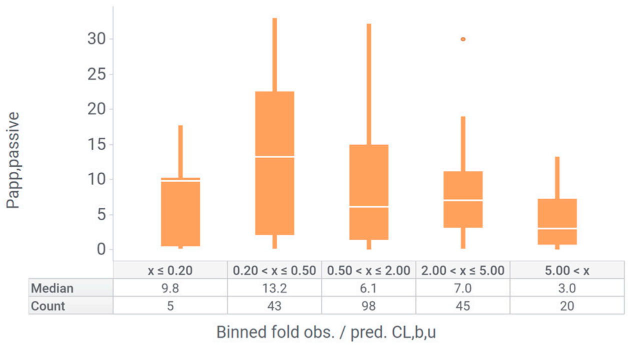


(**d**)

**Figure S4.** Box plots – Impact of CaCo-2 efflux ratio and passive permeability on clearance prediction accuracy. (**a**) and (**c**) Microsomes. (**b**) and (**d**) Hepatocytes. Obs.: observed, pred.: predicted, $CL_{b,u}$: unbound blood clearance, $P_{app, passive}$: apparent passive permeability, white line: median.

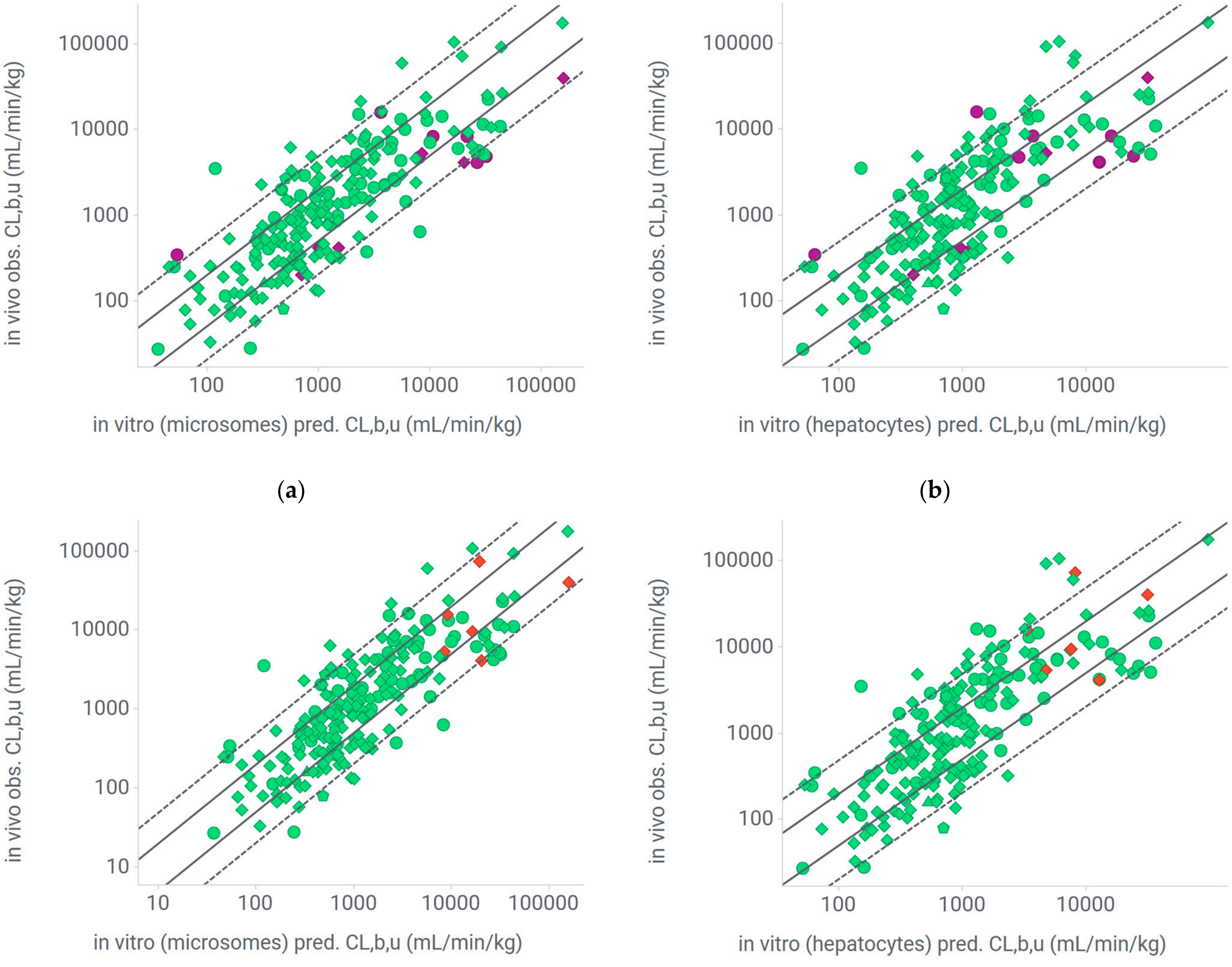


(**c**) (**d**)

**Figure S5.** IVIVE of unbound clearance in mice highlighting compounds with number of H-bond donors (HDon) >4 and containing VHL binder. (**a**) From microsomes, HDon >4 colored purple. (**b**) From hepatocytes HDon >4 colored purple. (**c**) From microsomes, VHL binder colored red. (**d**) From hepatocytes, VHL binder colored red. Pred.: predicted, obs.: observed, CL,b,u; unbound blood clearance; violet dots: containing >4 HDon, red dots: containing VHL binder, green dots: remaining compounds not obeying to respective criterion, bRo5 compounds; solid line: 2-fold, dashed line: 5-fold; triangles: acids, dots: bases; diamonds: neutrals, pentagons: zwitterions.

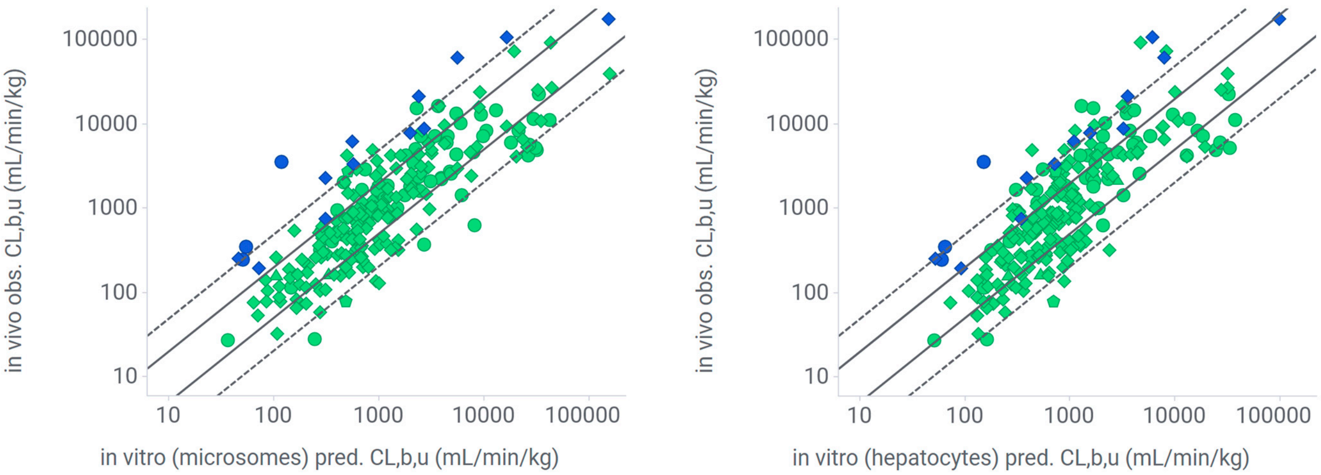


(**a**) (**b**)

**Figure S6.** IVIVE of unbound clearance in mice highlighting compounds with blood clearance above the liver blood flow. (**a**) From microsomes (**b**) From hepatocytes. Pred.: predicted, obs.: observed, $CL_{b,u}$; unbound blood clearance; green dots: clearance below the liver blood flow, blue dots: clearance above the liver blood flow; solid line: 2-fold, dashed line: 5-fold; triangles: acids, dots: bases; diamonds: neutrals, pentagons: zwitterions.

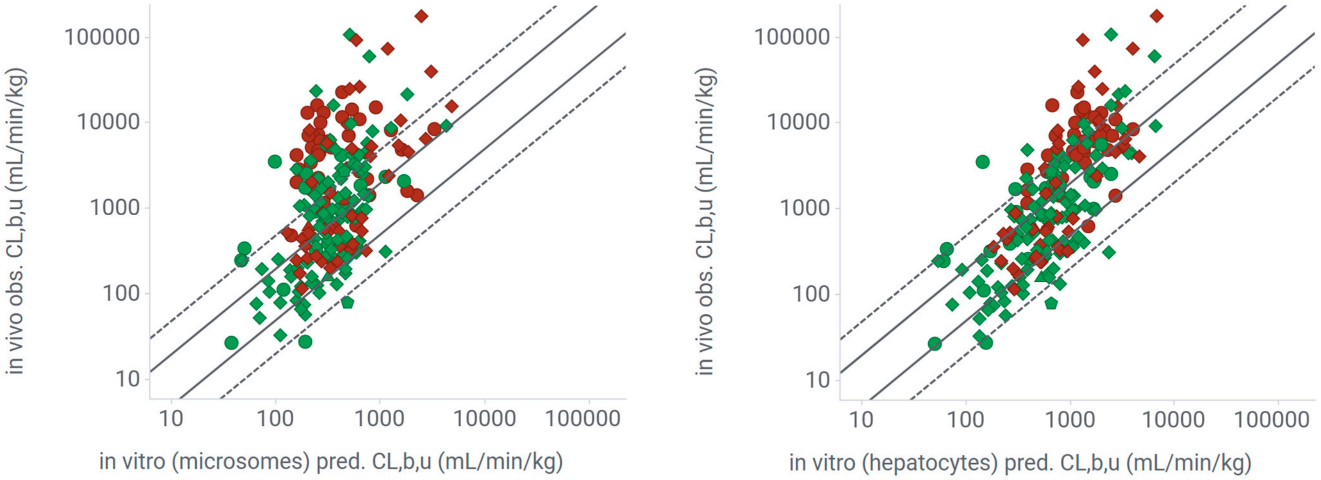


(**a**) (**b**)

**Figure S7.** IVIVE of unbound clearance in mice for rule-of-5 (Ro5) and beyond-Ro5 (bRo5) compounds using fraction unbound in the incubations estimated by the Kilford equation. (**a**) From microsomes (**b**) From hepatocytes. Pred.: predicted, obs.: observed, $CL_{b,u}$; unbound blood clearance; green dots: Ro5 compounds, red dots; bRo5 compounds; solid line: 2-fold, dashed line: 5-fold; triangles: acids, dots: bases; diamonds: neutrals, pentagons: zwitterions.

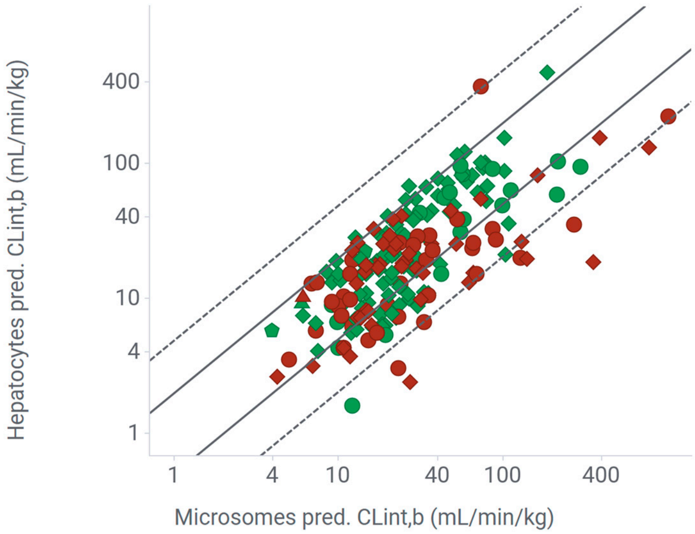


**Figure S8.** Correlation of intrinsic clearances predicted from hepatocytes *vs*. microsomes. Pred.: predicted, $CL_{int,b,u}$; unbound blood intrinsic clearance; green dots: Ro5 compounds, red dots; bRo5 compounds; solid line: 2-fold, dashed line: 5-fold; triangles: acids, dots: bases; diamonds: neutrals, pentagons: zwitterions.

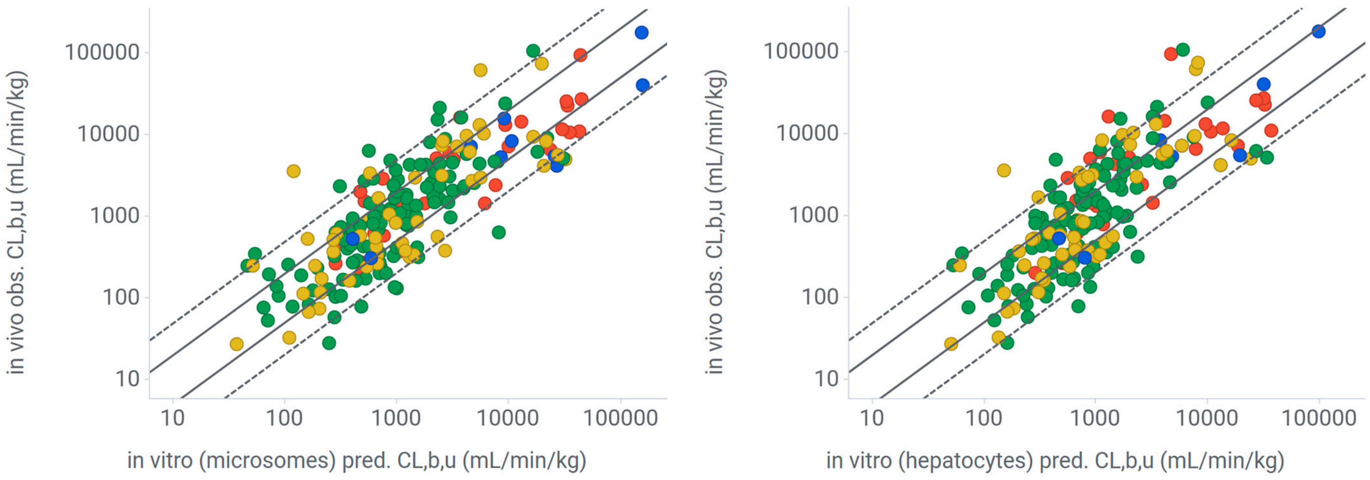


(**a**) (**b**)

**Figure S9. IVIVE of unbound clearance in mice highlighting that the different blood sampling techniques applied in *in vivo* PK studies have no impact on clearance extrapolation accuracy.** (**a**) From microsomes (**b**) From hepatocytes. Pred.: predicted, obs.: observed, $CL_{b,u}$; unbound blood clearance; green dots: retro-orbital, yellow dots: retro-orbital/saphenous vein, red dots: dorsal metatarsal vein, blue dots: submandibular; solid line: 2-fold, dashed line: 5-fold.
